# The predictive value of respiration parameters and the Glasgow Goma score for mortality of sepsis patients with acute respiratory distress syndrome: Insights from the MIMIC-IV database

**DOI:** 10.12669/pjms.41.11.13201

**Published:** 2025-11

**Authors:** Baiqing Ren, Muqiao Cheng, Yingxiu Liu, Zhile Li, Zhengshang Ruan

**Affiliations:** 1 Baiqing Ren Department of Intensive Care Unit, XinHua Hospital Affiliated with Shanghai Jiao Tong University, School of Medicine, Shanghai, People’s Republic of China; 2 Muqiao Cheng Department of Anesthesiology, XinHua Hospital Affiliated with Shanghai Jiao Tong University, School of Medicine, Shanghai, People’s Republic of China; 3 Yingxiu Liu Department of Intensive Care Unit, XinHua Hospital Affiliated with Shanghai Jiao Tong University, School of Medicine, Shanghai, People’s Republic of China; 4 Zhile Li Department of Intensive Care Unit, XinHua Hospital Affiliated with Shanghai Jiao Tong University, School of Medicine, Shanghai, People’s Republic of China; 5 Zhengshang Ruan Department of Intensive Care Unit, XinHua Hospital Affiliated with Shanghai Jiao Tong University, School of Medicine, Shanghai, People’s Republic of China

**Keywords:** Glasgow Coma Scale, Intensive Care Units, Mortality, Respiratory Distress Syndrome, Sepsis

## Abstract

**Background & Objective:**

Acute respiratory distress syndrome (ARDS) frequently complicates sepsis, leading to significant morbidity and mortality. This study aimed to identify factors that predict short-term (30-day) and long-term (one-year) mortality in ARDS patients and to develop robust predictive models.

**Methodology:**

This retrospective study, conducted from August 2024 to October 2024 in XinHua Hospital Affiliated with Shanghai Jiao Tong University, used data from the MIMIC database—specifically MIMIC IV 2.2—to identify sepsis patients who were diagnosed with ARDS within 24 hours of ICU admission. Univariate logistic regression was used to explore associations between respiratory parameters and the Glasgow coma scale (GCS) score (low group ≤ 12). Mortality at 30 days and one-year post-ICU admission was used as outcome measures. The dataset was balanced via synthetic minority over-sampling technique and split into training (70%) and validation (30%) sets. Variable selection was performed via the best subset, least absolute shrinkage and selection operator (LASSO), random forest, and boruta methods. Predictive models were developed and validated via calibration and decision curve analyses.

**Results:**

The cohort included 3,158 patients (58% female). Significant differences in PaCO_2_ levels were detected between 30-day survivors and non-survivors (p>0.05), but not detected at one year (p>0.05). More patients with low GCS scores died within one year (20.9%) than survivors (17.1%; p=0.01), but no such association was found for 30-day mortality (p>0.05). The predictive models perform well for predicating short-term and long-term mortality and had AUCs of 0.820 and 0.790, respectively.

**Conclusions:**

GCS scores were significantly associated with one-year mortality but not with 30-day mortality or with respiratory-related parameters. The developed predictive models demonstrated good performance. These findings aid in the treatment of ARDS patients with sepsis.

## INTRODUCTION

Sepsis is a life-threatening condition caused by a dysregulated host response to infection.^1^ Acute respiratory distress syndrome (ARDS) and acute respiratory failure (ARF) are common complications of sepsis and can lead to severe organ damage and even death.^2^ Additionally, the incidence rate of sepsis-associated encephalopathy (SAE) can reach 70%.^3^ A growing body of research shows that lung injury can promote brain damage through complex network processes that involve mechanical and chemical factors, as well as various inflammatory mediators.^4^ The impact of these lung and brain parameters on the short-term and long-term mortality of patients with sepsis is still unclear.

This study aimed to use the Medical Information Mart for Intensive Care-IV (MIMIC-IV) database of patients with sepsis complicated by ARDS to identify risk factors for short-term and long-term mortality and to establish a predictive model. Our results may help in estimating the risk of short- and long-term mortality in patients with sepsis accompanied by pulmonary conditions, detecting high-risk patients in a timely manner, and further improving the prognosis of this vulnerable group.

## METHODOLOGY

**Supplementary Fig.1:**
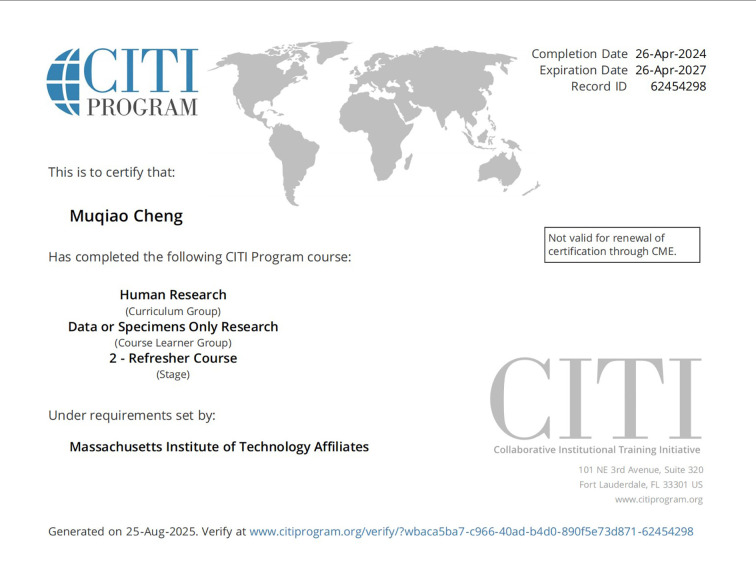
CITI completion certificate.

In the present study, conducted from August 2024 to October 2024 in XinHua Hospital Affiliated with Shanghai Jiao Tong University, the authors followed all applicable regulations, and all study data were sourced from the MIMIC database. First, the Institutional Review Boards (IRBs) of Beth Israel Deaconess Medical Center and the Massachusetts Institute of Technology granted the MIMIC-IV database a waiver of ethics approval and informed consent (Supplementary Fig.1). Second, the author (Record ID 62454298) obtained the necessary data-use permissions and completed Collaborative Institutional Training Initiative (CITI) certification prior to accessing the database.

### Inclusion criteria:

- The study population in MIMIC_IV comprised 9,064 adult patients diagnosed with sepsis (based on the diagnostic guidelines of sepsis-3) and with ARDS (PaO2/FiO2 ratio less than 300 mmHg), received by the ICU.

### Exclusion criteria:

- Pulmonary neoplasms.
- Neurological disorders.
- More than 80% missing data.

**Supplementary Fig.2:**
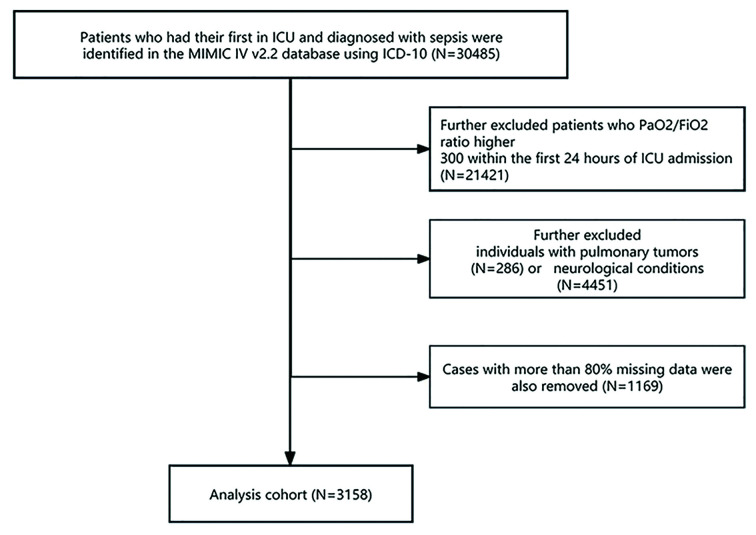
Flowchart illustrating the screening criteria for patient inclusion in this study.

A final sample of 3,158 patients was included in the study (Supplementary Fig.2). Patients were stratified into 30-day survivors and non-survivors, and, in parallel, into one-year survivors and non-survivors, according to vital status at 30 days and one year, respectively. Only data from the initial ICU admission were considered for patients admitted multiple times.

### Data collection and analysis:

Demographics, medical history, vital signs, laboratory tests, and scores within the first 24 hours post-ICU admission were recorded, and cases with more than 80% missing data were removed. Univariate logistic regression was used to examine the correlation between GCS scores and ventilator parameters, then assessed the associations of GCS scores with 30 days and one-year mortality, and subsequently constructed predictive models for mortality at both time points.

### Statistical analysis:

Continuous variables were described using mean (standard deviation [SD]) or median (interquartile range [IQR]). Group comparisons were performed using the Mann-Whitney U test or Student’s t-test. Categorical variables were presented as frequencies and percentages (%), and group comparisons were conducted using Fisher’s exact test or the chi-square test. Univariate logistic regression was used to explore the relationships between variables.

### Predictive model development and Validation:

Variables with more than 20% missing data were excluded from the MIMIC database analysis. To improve model accuracy, data underwent SMOTE balancing to equalize the proportions of deceased and surviving patients. The cohort was then randomly divided into a training set (70%) and a validation set (30%). All modeling procedures were performed separately for 30-day and one-year mortality. The selection methods included best subset, least absolute shrinkage and selection operator (LASSO) regression, random forest (RF), and Boruta. The selected variables were included in a multivariable logistic regression model. The best models were chosen on the basis of the BIC, AIC, and ROC analyses, with performance illustrated through nomograms, calibration curves, Hosmer–Lemeshow (HL) testing, and decision curve analysis (DCA). Model robustness was assessed via unbalanced datasets and across patient groups with initial 24-hour SOFA scores ≥2. Statistical significance was set at *p* < 0.05. Data analysis was conducted via R software (version 4.4.0).

## RESULTS

### Baseline and association analysis:

Baseline characteristics according to 30-day mortality are presented in Table-I. Corresponding data stratified by one-year survival status are shown in Table-II. No relationships were detected between the GCS score and respiratory parameters (*p* > 0.05) (Supplementary Table-I). The percentage of patients with GCS scores ≤12 was greater in the one-year mortality group (20.9%) than in the long-term survival group (17.1%, *p* = 0.01), but no difference was found in the 30-day mortality group (*p* > 0.05).

**Table-I:** Baseline characteristics of patients stratified by 30-day mortality status.

Characteristic	30-day mortality status				p-value
	Overall (N=3158)	Survived (N=2423, 76.7%)	Dead (N=735, 23.3%)		
Gender No.(%)					
Male	1326 (42.0)	983 (40.6)	343 (46.7)	0.004	
Female	1832 (58.0)	1440 (59.4)	392 (53.3)		
Length of hospital M[IQR]	9.23 [5.39, 15.72]	10.30 [6.51, 17.11]	4.97 [1.71, 10.64]	<0.001	
Age M[IQR]	68.10 [57.03, 78.57]	67.06 [55.80, 77.08]	72.07 [61.00, 82.41]	<0.001	
Length of ICU M[IQR]	3.98 [2.02, 7.90]	4.05 [2.16, 7.89]	3.72 [1.43, 7.93]	<0.001	
Weight M[IQR]	80.00 [67.90, 95.53]	81.00 [68.50, 96.03]	77.30 [65.00, 93.93]	<0.001	
Heart rate M[IQR]	72.00 [62.00, 83.00]	71.00 [62.00, 81.00]	76.00 [62.00, 89.00]	<0.001	
Respiration rate M[IQR]	12.00 [10.00, 15.00]	12.00 [10.00, 14.50]	14.00 [10.00, 17.00]	<0.001	
MAP M[IQR]	56.67 [50.67, 62.00]	57.50 [52.33, 63.00]	52.33 [44.33, 59.00]	<0.001	
SpO_2_ M[IQR]	92.00 [89.00, 94.00]	92.00 [90.00, 95.00]	90.00 [82.00, 93.00]	<0.001	
PH M[IQR]	7.30 [7.22, 7.36]	7.31 [7.24, 7.36]	7.24 [7.13, 7.34]	<0.001	
PaO_2_ M[IQR]	81.00 [68.00, 100.00]	83.00 [70.00, 103.00]	73.00 [61.00, 90.00]	<0.001	
PaCO_2_ M[IQR]	35.00 [31.00, 40.00]	35.00 [31.00, 40.00]	34.00 [29.00, 40.00]	0.007	
FiO_2_ M[IQR]	50.00 [40.00, 50.00]	50.00 [40.00, 50.00]	50.00 [40.00, 70.00]	<0.001	
Ratio of PaO_2_:FiO_2_ M[IQR]	156.00 [101.49, 212.00]	166.00 [111.00, 220.00]	120.00 [78.17, 180.00]	<0.001	
BE M[IQR]	-4.00 [-8.00, 0.00]	-3.00 [-6.00, 0.00]	-8.00 [-13.00, -1.00]	<0.001	
Lactate M[IQR]	1.40 [1.00, 2.10]	1.30 [1.00, 1.70]	2.10 [1.40, 4.05]	<0.001	
White blood cell M[IQR]	10.40 [7.20, 14.40]	10.20 [7.30, 13.70]	11.70 [6.60, 17.10]	<0.001	
Hemoglobin M[IQR]	9.60 [8.30, 11.10]	9.60 [8.30, 11.20]	9.40 [8.10, 11.00]	0.014	
Platelet M[IQR]	153.00 [103.00, 219.00]	156.00 [109.25, 218.00]	140.00 [74.00, 224.00]	<0.001	
Red blood cell M[IQR]	3.18 [2.74, 3.72]	3.19 [2.77, 3.74]	3.13 [2.66, 3.64]	0.001	
Creatinine M[IQR]	0.90 [0.70, 1.40]	0.90 [0.70, 1.20]	1.40 [0.90, 2.30]	<0.001	
BUN M[IQR]	19.00 [13.00, 31.00]	17.00 [12.00, 26.00]	31.00 [20.00, 46.00]	<0.001	
SOFA M[IQR]	2.00 [1.00, 4.00]	2.00 [0.00, 4.00]	3.00 [1.00, 5.50]	<0.001	
Intubation at first day of ICU No.(%)					
No	250 (7.9)	194 (8.0)	56 (7.6)	0.815	
Yes	2908 (92.1)	2229 (92.0)	679 (92.4)		
GCS No.(%)					
<=12	581 (18.4)	434 (17.9)	147 (20.1)	0.192	
13-15	2574 (81.6)	1988 (82.1)	586 (79.9)		

**ICU =** intensive care unit, **MAP =** mean arterial pressure, **pH=** pondus hydrogenii, **BE =** base excess, **BUN =** blood urea nitrogen, **SOFA =** sequential organ failure assessment, **GCS =** Glasgow Coma Scale.

**Table-II:** Baseline characteristics of patients stratified by one-year mortality status.

Characteristic	one-year mortality status			p-value
	Overall (N=3158)	Survived (N=2068, 65.5%)	Dead (N=1090, 34.5%)	
Gender No.(%)				
Male	1326 (42.0)	823 (39.8)	503 (46.1)	0.001
Female	1832 (58.0)	1245 (60.2)	587 (53.9)	
Length of hospital M[IQR]	9.23 [5.39, 15.72]	9.88 [6.25, 15.89]	7.97 [2.99, 14.97]	<0.001
Age M[IQR]	68.10 [57.03, 78.57]	65.79 [54.63, 75.76]	73.78 [61.41, 82.38]	<0.001
Length of ICU M[IQR]	3.98 [2.02, 7.90]	3.89 [2.10, 7.31]	4.25 [1.85, 9.00]	0.196
Weight M[IQR]	80.00 [67.90, 95.53]	82.00 [70.00, 97.15]	76.10 [63.80, 92.40]	<0.001
Heart rate M[IQR]	72.00 [62.00, 83.00]	71.00 [62.00, 81.00]	74.00 [62.00, 86.00]	<0.001
Respiration rate M[IQR]	12.00 [10.00, 15.00]	12.00 [10.00, 14.00]	13.00 [10.00, 16.00]	<0.001
MAP M[IQR]	56.67 [50.67, 62.00]	57.83 [52.67, 63.33]	54.00 [46.42, 59.33]	<0.001
SpO_2_ M[IQR]	92.00 [89.00, 94.00]	92.00 [90.00, 95.00]	91.00 [85.75, 94.00]	<0.001
PH M[IQR]	7.30 [7.22, 7.36]	7.31 [7.24, 7.36]	7.26 [7.16, 7.35]	<0.001
PaO_2_ M[IQR]	81.00 [68.00, 100.00]	84.00 [70.00, 103.00]	75.00 [63.00, 92.00]	<0.001
PaCO_2_ M[IQR]	35.00 [31.00, 40.00]	35.00 [31.00, 39.00]	35.00 [30.00, 41.00]	0.317
FiO_2_ M[IQR]	50.00 [40.00, 50.00]	50.00 [40.00, 50.00]	50.00 [40.00, 60.00]	<0.001
Ratio of PaO_2_:FiO_2_ M[IQR]	156.00 [101.49, 212.00]	166.00 [112.86, 218.00]	134.64 [85.71, 196.92]	<0.001
BE M[IQR]	-4.00 [-8.00, 0.00]	-3.00 [-6.00, 0.00]	-6.00 [-12.00, -0.25]	<0.001
Lactate M[IQR]	1.40 [1.00, 2.10]	1.30 [1.00, 1.70]	1.80 [1.20, 3.30]	<0.001
White blood cell M[IQR]	10.40 [7.20, 14.40]	10.20 [7.30, 13.60]	11.20 [6.82, 16.20]	0.001
Hemoglobin M[IQR]	9.60 [8.30, 11.10]	9.60 [8.30, 11.20]	9.40 [8.10, 11.00]	0.005
Platelet M[IQR]	153.00 [103.00, 219.00]	156.00 [111.00, 217.00]	147.00 [83.00, 227.00]	<0.001
Red blood cell M[IQR]	3.18 [2.74, 3.72]	3.20 [2.77, 3.74]	3.14 [2.67, 3.69]	0.001
Creatinine M[IQR]	0.90 [0.70, 1.40]	0.90 [0.70, 1.20]	1.30 [0.80, 2.10]	<0.001
BUN M[IQR]	19.00 [13.00, 31.00]	16.00 [12.00, 24.00]	29.00 [18.00, 43.00]	<0.001
SOFA M[IQR]	2.00 [1.00, 4.00]	2.00 [0.00, 4.00]	3.00 [1.00, 5.00]	<0.001
Intubation at first day of ICU No.(%)				
No	250 (7.9)	152 (7.4)	98 (9.0)	0.111
Yes	2908 (92.1)	1916 (92.6)	992 (91.0)	
GCS No.(%)				
<=12	581 (18.4)	354 (17.1)	227 (20.9)	0.010
13-15	2574 (81.6)	1713 (82.9)	861 (79.1)	

**ICU =** intensive care unit, **MAP =** mean arterial pressure, **pH=** pondus hydrogenii, **BE =** base excess, **BUN =**blood urea nitrogen, **SOFA =** sequential organ failure assessment, **GCS =** Glasgow Coma Scale

**Supplementary Table-I:** The relationship between Glasgow Coma Scores and respiratory parameters.

Variables	Overall(N=3158)	GCS<=12(N=581, 18.5%)	12<GCS<=15(N=2574,81.5%)	p-value
Respiratory rate ***M(IQR)***	12.00 [10.00, 15.00]	12.00 [10.00, 15.00]	12.00 [10.00, 15.00]	0.224
SpO_2_ ***M(IQR)***	92.00 [89.00, 94.00]	92.00 [88.00, 94.00]	92.00 [89.00, 94.00]	0.184
pH ***Mean (SD)***	7.28 (0.12)	7.27 (0.12)	7.28 (0.12)	0.094
PaO_2_ ***M(IQR)***	81.00 [68.00, 100.00]	79.00 [66.00, 97.00]	81.00 [68.00, 101.00]	0.069
PaCO_2_ ***M(IQR)***	35.00 [31.00, 40.00]	35.00 [31.00, 40.00]	35.00 [31.00, 40.00]	0.497
FiO_2_ ***M(IQR)***	50.00 [40.00, 50.00]	50.00 [40.00, 50.00]	50.00 [40.00, 50.00]	0.325
PaO_2_/FiO_2_ ratio ***M(IQR)***	156.00 [101.49, 212.00]	151.67 [100.00, 206.25]	158.00 [102.00, 214.00]	0.177
BE ***Mean (SD)***	-4.63 (6.43)	-4.63 (6.33)	-4.63 (6.45)	0.992
Lactate ***Mean (SD)***	1.96 (1.92)	2.00 (1.77)	1.95 (1.93)	0.607
PEEP ***M(IQR)***	5.00 [5.00, 5.00]	5.00 [5.00, 5.00]	5.00 [5.00, 5.00]	0.713
Ventilator respiratory rate total ***M(IQR)***	16.00 [14.00, 19.00]	16.00 [14.00, 19.00]	16.00 [14.00, 18.00]	0.855
Ventilator respiratory rate spontaneous **M(IQR)**	0.00 [0.00, 0.00]	0.00 [0.00, 0.00]	0.00 [0.00, 0.00]	0.408
Ventilator minute volume ***M(IQR)***	7.39 [6.20, 8.69]	7.50 [6.20, 8.78]	7.30 [6.20, 8.60]	0.295
Ventilator tidal volume observed ***M(IQR)***	413.00 [340.00, 480.00]	420.50 [330.00, 490.00]	411.00 [340.84, 479.00]	0.443
Ventilator FiO_2_ ***M(IQR)***	40.00 [40.00, 50.00]	40.00 [40.00, 50.00]	40.00 [40.00, 50.00]	0.210

pH, pondus hydrogenii; BE, base excess; PEEP, positive end-expiratory pressure. The results indicate there was no relationships between Glasgow Coma Scores and respiratory parameters including blood gas analysis, respiratory parameters and ventilator-related parameters.

### Model construction:

**Fig.1:**
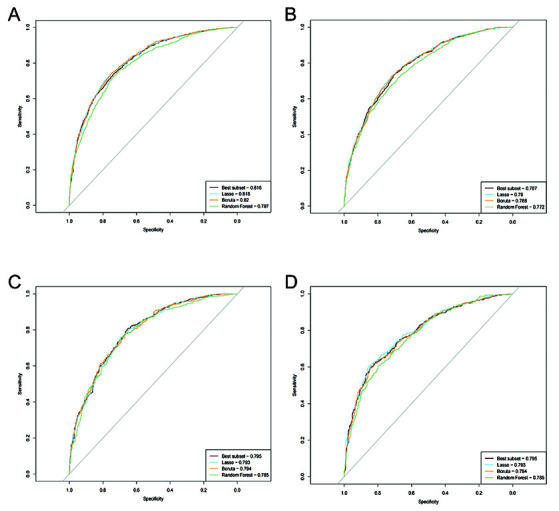
ROC curves and AUC values for different models in predicting 30-day (Panels a and c) and one-year (Panels b and d) mortality. **a) and b):** ROC curves and AUC values for different models in the training set. **c) and d):** ROC curves and AUC values for different models in the test set.

We evaluated multiple models for predicting 30-day and one-year mortality using different variable-selection approaches. Fig.1 presents the ROC curves and corresponding AUC values for the training (Fig.1 a & b) and test sets (Fig.1 c & d); panels a and c depict 30 days mortality, whereas panels b and d depict one-year mortality. The models were assessed on the basis of the AIC, BIC, and AUC (results represented in Table-III).

**Table-III:** The corresponding evaluation indices (AIC, BIC, etc.) are constructed for building models via different filtering variable methods to reflect the goodness of fit and complexity.

Target	Method	Index					
		AIC^1^	BIC^1^	AUC^1^	Cut-off	Sensitivity	Specificity
***30 days mortality***	Best subset	2341.53	2404.24*	0.816	0.477	0.733	0.744
	LASSO^2^	2335.9	2404.32	0.818	0.494	0.758*	0.737
	Boruta	2328.94*	2408.76	0.820*	0.478	0.742	0.749
	RF^2^	2422.40	2462.31	0.797	0.463	0.703	0.759*
***One-year mortality***	Best subset	2480.33	2543.05	0.787	0.471	0.706	0.733*
	LASSO^2^	2469.37*	2549.19	0.790*	0.479	0.716	0.729
	Boruta	2474.31	2542.73*	0.788	0.481	0.722*	0.723
	RF^2^	2535.41	2586.73	0.772	0.473	0.684	0.715

1 **AIC:** Akaike information criterion; **BIC:** Bayesian information criterion; **AUC:** area under the curve.

2 **LASSO:** least absolute shrinkage and selection operator; **RF:** random forest.

* Denotes the best evaluation index of each model.

**Fig.2:**
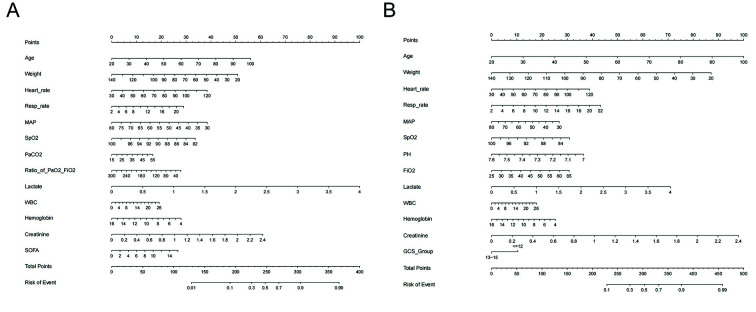
Nomograms for Predicting 30-day and one-year Mortality. Panel **a)** depicts the nomogram for predicting 30-day mortality, while Panel **b)** illustrates the nomogram for predicting one-year mortality.

We selected the Boruta model for 30-day mortality, which achieved an AUC of 0.820, a sensitivity of 0.742, and a specificity of 0.749. The LASSO model for one-year mortality had an AUC of 0.790, a sensitivity of 0.716, and a specificity of 0.729. Fig.2 illustrates the variables selected for 30-day mortality prediction (Fig.2a) and one-year mortality prediction (Fig.2b). Notable odds ratios (ORs) for 30-day mortality included lactate (OR: 2.63) and Cre (OR: 2.66). For one-year mortality, significant ORs included pH (OR: 0.17), lactate (OR: 1.69), Cre (OR: 3.35), and GCS (OR: 0.74).

### Model evaluation and validation:

**Fig.3:**
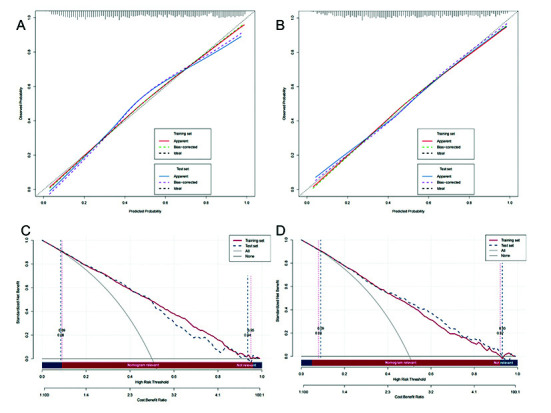
Calibration curves and DCA curves for predicting 30-day and one-year mortality. Panels **a)** and **c)** present the results for predicting 30-day mortality, whereas Panels **b)** and **d)** illustrate the results for predicting one-year mortality. The numbers on the curves indicate the corresponding risk threshold values.

**Fig.4:**
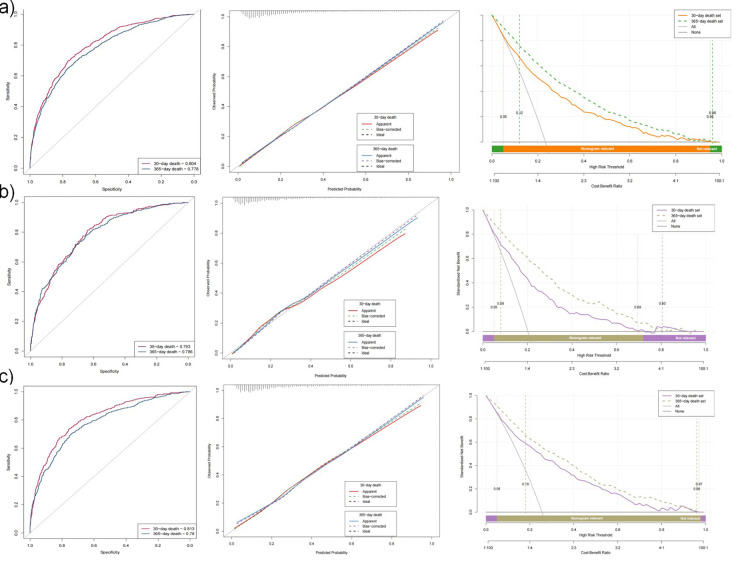
Model Performance in Predicting 30-day and One-year Mortality. These figures represent the ROC curves, AUC results, calibration curves, and DCA curves for predicting 30-day and one-year mortality across different datasets and patient subgroups. Specifically, **a)** illustrates model performance in an unbalanced dataset without SMOTE, **b)** focuses on patients with SOFA scores less than 2, and **c)** examines patients with SOFA scores of 2 or higher.

The calibration curves showed good fit (Fig.3, a & b). The Hosmer–Lemeshow test yielded *p-values* of 0.517 and 0.385 for the 30-day and one-year models, respectively. Decision curve analysis (DCA) plots (Fig.3, c & d) indicated substantial net benefit across a relevant range of threshold probabilities, highlighting the predictive capabilities of the models. To validate the predictive performance of our models, additional analyses were conducted on different subpopulation datasets and unbalanced data (Fig.4a). Patients were stratified by their initial SOFA score upon ICU admission (SOFA score <2 vs. ≥2), and models were validated within these subgroups. Results are presented in Supplementary Table-II, Fig.4b (SOFA<2), and Fig.4c (SOFA≥2). These analyses highlight the robustness and generalizability of our models.

**Supplementary Table-II:** Model performance evaluation on imbalanced data across different populations.

Data	Target	Index				
		AUC^1^	Cut-off	Sensitivity	Specificity	Hosmer-Lemeshow test
**No-SMOTE^2^**	30-day	0.804	0.244	0.766	0.699	0.357
	One-year	0.778	0.370	0.771	0.649	0.917
**SOFA^2^< 2**	30-day	0.793	0.163	0.640	0.812	0.137
	One-year	0.786	0.260	0.645	0.783	0.862
**SOFA^2^ ≥ 2**	30-day	0.813	0.250	0.756	0.737	0.453
	One-year	0.786	0.260	0.645	0.783	0.699

1 AUC, area under curve.

2 SMOTE, synthetic minority over-sampling technique; SOFA, sequential organ failure assessment.

## DISCUSSION

Sepsis is a severe multisystem disorder with high mortality. Our previous work elucidated the link between sepsis-associated AKI and mortality^5^, and the present study further explores factors related to SAE and establishes a relevant model. Our study revealed that the 30-day and one-year mortality rates for sepsis patients with ARDS were 23.27% and 34.52%, respectively. Our study also showed that 30 days mortality was associated with a higher respiratory rate and lower oxygen saturation, pH, PaO_2_, PaCO_2_, oxygenation index. The pulmonary-brain axis has received increasing attention in recent years.^6^ In 64% of all sepsis cases, the infection leading to sepsis originates in the lungs.^1^ Inflammatory factors from chronic inflammation in asthma can impact brain regions, leading to neurological symptoms.^7^ Animal experiments also confirmed that lung infections can damage brain nerves.^8^ For now, the only reliable clinical measurement of brain function was the GCS score.^9^-^11^ Interestingly, we found no correlations between the GCS score and respiration parameters, possibly because of indirect pathways such as those involving inflammatory factors.^12^

More intriguingly, our findings demonstrated that several factors differed significantly between patients who died within one year of ICU admission and those who died within 30 days. Specifically, PaCO_2_ differed markedly between the 30-day non-survivors and longer-term survivors. Nevertheless, PaCO_2_ was not retained in the final predictive model for one-year mortality, suggesting that its prognostic relevance may be confined to short-term outcomes. In contrast, the severity of neurologic injury emerged as a pivotal determinant of long-term survival. Lu et al. found that mechanical ventilation parameters influence patients’ prognosis, which is not supported by the findings of our study.^13^ Notably, their research was a single-center study with a small cohort that employed regression analysis. These difference might account for variations in medical standards across different regions. This discrepancy between the two studies warrants further verification through future randomized controlled trials.

We also found that higher GCS scores were significantly associated with lower long-term (one-year) mortality. As reported by Reynolds JC et al. and Malhotra AK et al., long-term mortality in patients with ARDS is associated with a lower GCS; a similar finding was observed in our cohort of septic patients with ARDS.^14^,^15^ COVID-19, flu, or bacterial pneumonia were shown to have a long-term impact on the cognitive function and concentration of patients and the impairments in cognitive function and concentration can lead to errors and delays in judgments related to hazard avoidance.^16^ Meanwhile, these defects affect multiple aspects of patients’ work and daily lives. Such consequences may cause to the increased long-term mortality rate among patients with ARDS.

Prior studies have demonstrated that acute respiratory distress syndrome (ARDS) occurs frequently in patients with severe acute brain injury (SABI) and portends worse neurological outcomes.^17^ However, three major conflicts exist between lung-protective ventilation recommended for ARDS and the cerebral-protective strategies required for SABI.^1^,^18^ These conflicts may further increase mortality in sepsis. In the current cohort of mechanically ventilated septic patients we observed that respiratory parameters were not correlated with the Glasgow Coma Scale (GCS). This finding indirectly suggests that the relationship between GCS and mortality is independent of ventilatory support, a result that appears to diverge from earlier reports.^19^ Consequently, reliance on GCS alone as a surrogate for cerebral function may be insufficient.

**Supplementary Fig.3:**
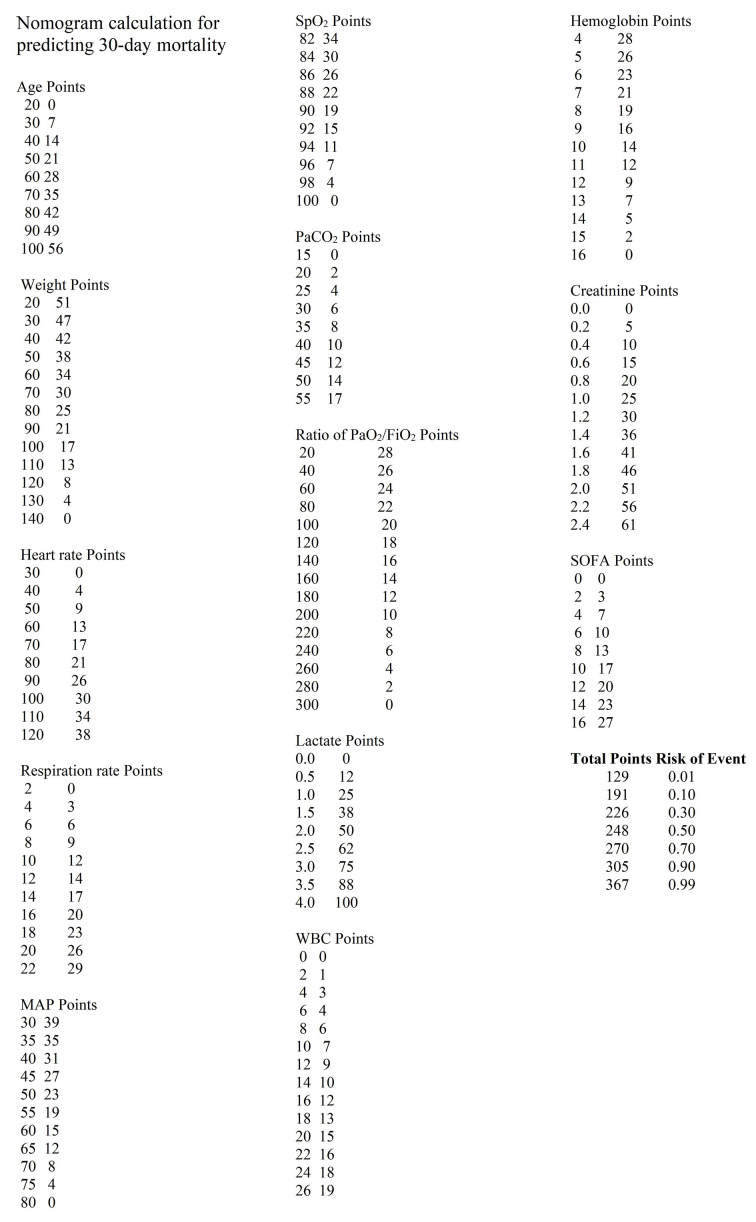
Nomogram calculation for predicting 30 days mortality.

**Supplementary Fig.4:**
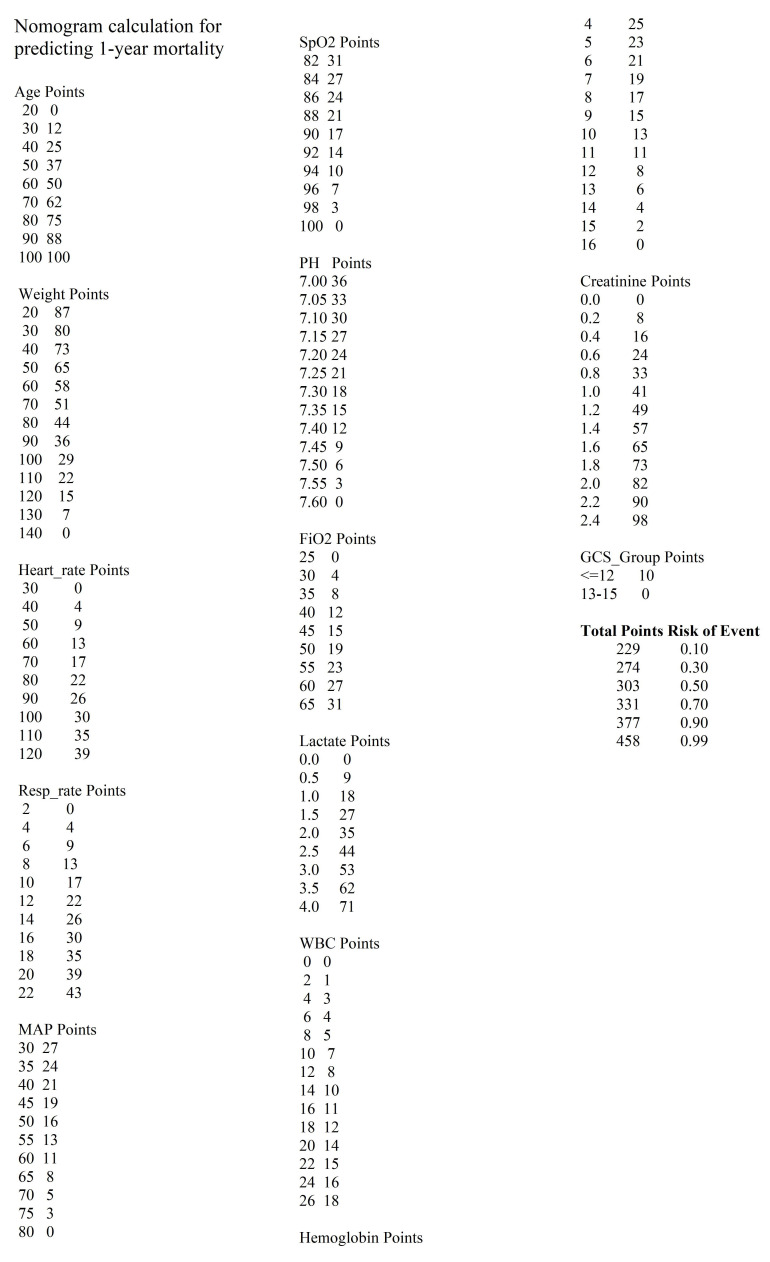
Nomogram calculation for predicting 1-year mortality.

To predict 30 days and one-year mortality, we developed two robust models for clinical use (The detailed calculation method can be found in Supplementary Fig.3 and 4). Jiyeon Roh and colleagues previously constructed a nomogram to estimate long-term mortality in sepsis (including septic shock) using data from 446 patients (60.8% male; median age, 71 years) treated for ≥ 3 years at a university-affiliated tertiary care hospital.^20^ Multivariate analysis identified age ≥ 65 years, BMI < 18.5 kg/m², hematologic malignancy, and mechanical ventilation as predictors for both the 180- and 365-day models. The corresponding AUCs were 0.713 (95% CI, 0.668–0.758) and 0.697 (95% CI, 0.651–0.743), which are lower than those achieved by our current models.

Our study provides short-term and long-term prediction models as well as practical predictive tools for patients with ARDS caused by sepsis. Notably, the factors incorporated into the modeling are all actual predictors of ARDS patients’ prognosis, which offers important basis for future RCTs and mechanism-related studies. Meanwhile, with more studies suggesting that the brain is an easily overlooked yet critical and overloaded organ in sepsis, our study reveals that a low GCS score is associated with poor long-term prognosis in patients with sepsis complicated by ARDS—this provides important insights for future research.

### Limitations

First, we excluded more than 80% of the cases with missing data during the data processing phase, which may have introduced potential bias. Second, we choose only the GCS score as a parameter for brain function, but there might many other potential parameters can reflect brain function. Third, despite the successful internal validation of our models, the lack of external data hinders additional validation.

## CONCLUSIONS

This study revealed that in sepsis patients with ARDS, higher GCS scores were significantly associated with lower mortality one year after admission to the ICU. Predictive models for both short-term and long-term mortality in patients were constructed and have demonstrated good predictive value.

### Authors’ contributions:

**ZR**: Designed the study, participated in data collection, guided the research, and reviewed the manuscript. This author is also responsible and accountable for the accuracy and integrity of the work.

**BR:** Literature search, data analysis and finalized the manuscript and

**MC:** Literature search, Drafted the manuscript.

**YL:** Assisted in data collection and analysis and manuscript revision.

**ZL:** Participated in literature search, writing and revising the manuscript.

All authors have contributed equally to the manuscript and read and approved the final version.
